# Cervical and endometrial cancers of intestinal origin diagnosed 18 years after vaginal replacement with a sigmoid colon segment: a case report and review of the literature

**DOI:** 10.3389/fonc.2022.912236

**Published:** 2022-07-28

**Authors:** Xiao-fei Sun, Xin-xin Miao, Bao-xuan Li, Yu Wang

**Affiliations:** Department of Obstetrics and Gynecology, Shengjing Hospital of China Medical University, Shenyang, China

**Keywords:** neovagina, vaginal agenesis, adenocarcinoma, sigmoid colpoplasty, sigmoid colon neovagina

## Abstract

**Background:**

Sigmoid colpoplasty is a surgical method for the treatment of vagina agenesis. Malignant tumors of neovaginas derived from sigmoid colons are rare.

**Case presentation:**

We report a 33-year-old woman who underwent sigmoid colpoplasty for vaginal agenesis and presented 18 years later with vaginal bleeding. Examination revealed cancer of the neovagina with involvement of the cervix and endometrium. The patient was administered four cycles of chemotherapy because she refused surgery.

**Conclusions:**

Patients with a history of colpoplasty should undergo long-term comprehensive testing after reconstruction, including regular gynecological, colposcopic, and gastrointestinal examinations. In patients with cancer of the neovagina, a comprehensive treatment plan should be developed in consultation with gynecologists and surgeons. There is no standard treatment, although surgery plus chemotherapy or radiotherapy appears to be effective.

## Introduction

Vaginal agenesis is a rare disease worldwide. The absence of a vagina causes the rectum to be in close proximity to the bladder and urethra. The first-line treatment is vaginal elongation by dilation, whereas colpoplasty is appropriate for patients who have failed dilation or who have voluntarily opted for surgery. In patients with a uterus who can menstruate, it is possible to attach the cervical opening to alternative tissues and to artificially restore vaginal and uterine functions.

There are many types of colpoplasty; one method is to use the sigmoid colon as a substitute for the vagina. Because of the elasticity of the intestinal wall, there is no need to use a mold for postoperative dilation; moreover, an artificial vagina formed with the bowel has glandular secretions that provide lubrication, allowing for satisfactory sexual activity. However, sigmoid colpoplasty may cause various bowel-related complications, such as intestinal obstruction, anastomotic fistula, vaginal stenosis, artificial vaginal prolapse, colitis, and hereditary polyposis. A rarer complication is the development of a malignant neoplasm of intestinal origin.

Herein, we report a rare case of a patient who developed a malignancy in the neovagina that involved the endometrium and cervix 18 years after she underwent sigmoid colpoplasty.

## Case presentation

**Figure 1:**
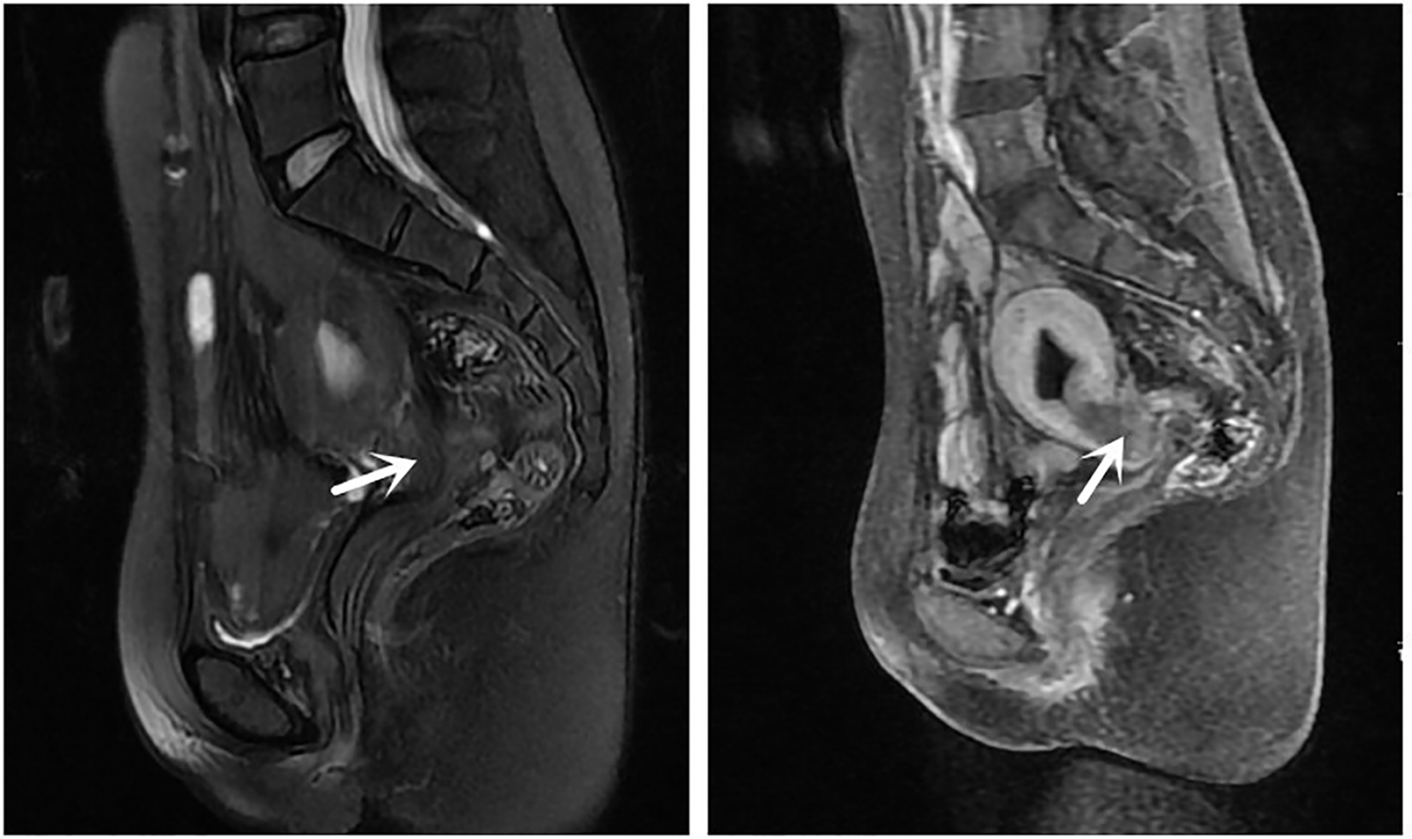
Magnetic resonance images showing an irregular mass in the cervical area. The irregular mass shadow (3.8 × 1.2 × 2.0 cm) is visible with a thickened and prominent cervical posterior wall. There is suspected penetration of vaginal posterior wall and involvement of the rectum (arrow).

**Figure 2:**
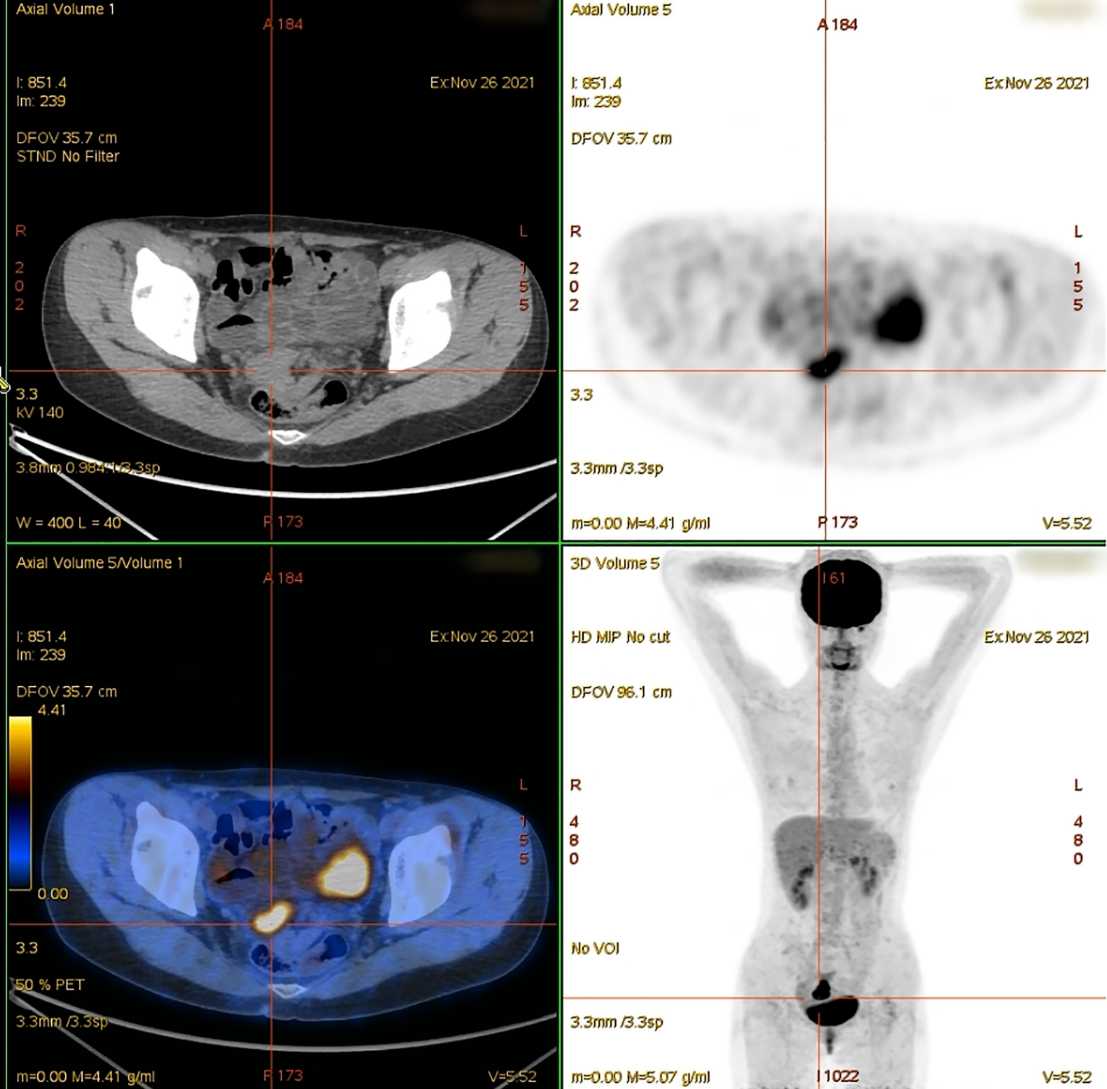
Positron emission tomography/computed tomography, showing a mass with fluorodeoxyglucose (FDG) uptake in the cervix (maximum standardized uptake: 10.49).

The patient was a 33-year-old married woman who underwent sigmoid colpoplasty at our hospital in 2003 (when she was 15 years old) for vaginal agenesis(Classified as U0C4V4 according to 2013 ESHRE-ESGE (1)); she had a normally developed uterus. A few months after surgery, the patient was found to have a purulent pelvic mass and underwent another surgical procedure to remove it. The patient married at the age of 27 years and engaged in sexual activity twice a week, but was unable to conceive spontaneously. She had no history of smoking or alcohol consumption, and there was no family history of any hereditary diseases. The patient presented with lower abdominal pain and vaginal spotting that had persisted for 1 month at the time of consultation; gynecological examination indicated partial cervicovaginal stenosis and an inability to expose the cervix. Transvaginal ultrasonography indicated fluid collection in the upper uterine cavity measuring approximately 1.3 × 2.9 × 0.8 cm and an endometrial thickness of approximately 1.0 cm in the lower uterine cavity. Enhanced pelvic magnetic resonance imaging indicated an 3.8 × 1.2 × 2.0 cm irregular mass in the lower uterine cavity-cervical region with a prominent thickening of the posterior cervical wall; penetration of the posterior vaginal wall with rectal involvement was also suspected (**Figure 1**). Laboratory examination revealed a carcinoembryonic antigen level of 160.8 ng/mL and cancer antigen 19-9 level of 722.7 U/mL. Gastroscopy was subsequently performed, and pathological examination suggested mild inflammation of the intestinal tract. ^18^F-fluorodeoxyglucose positron emission tomography/computed tomography indicated increased fluorodeoxyglucose metabolism in the cervix and endometrium with involvement of the lower uterine corpus and vagina (**Figure 2**). Behind the cervix and vagina, an irregular mass with low fluorodeoxyglucose metabolism and poor demarcation from the adjacent rectum and sigmoid colon was observed.

**Figure 3:**
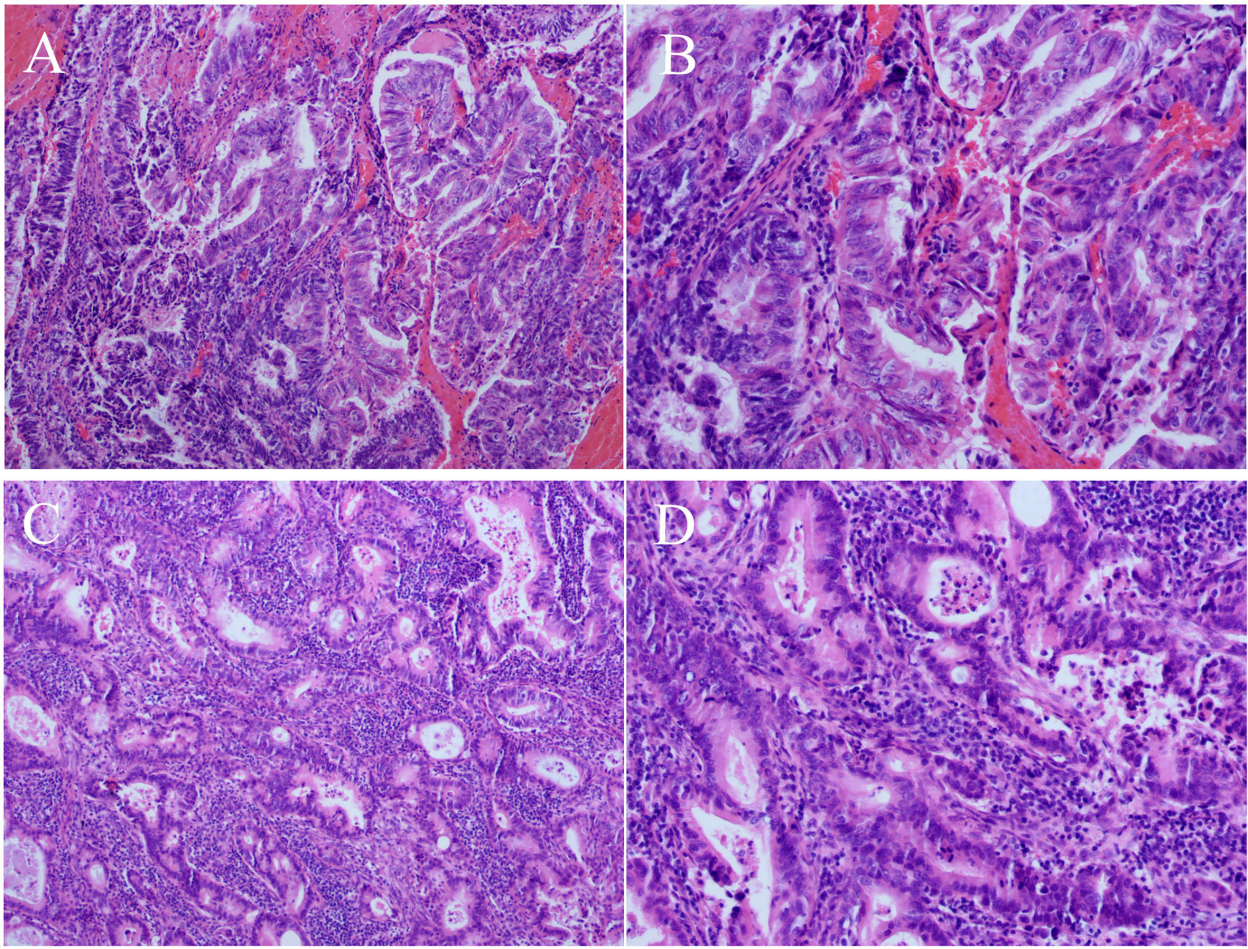
Histopathological findings (hematoxylin-eosin staining). Well-differentiated adenocarcinoma of the cervix **(A)**, 100×; **(B)**, 200× and well-differentiated adenocarcinoma of the endometrium **(C)**, 100×; **(D)**, 200×.

**Figure 4:**
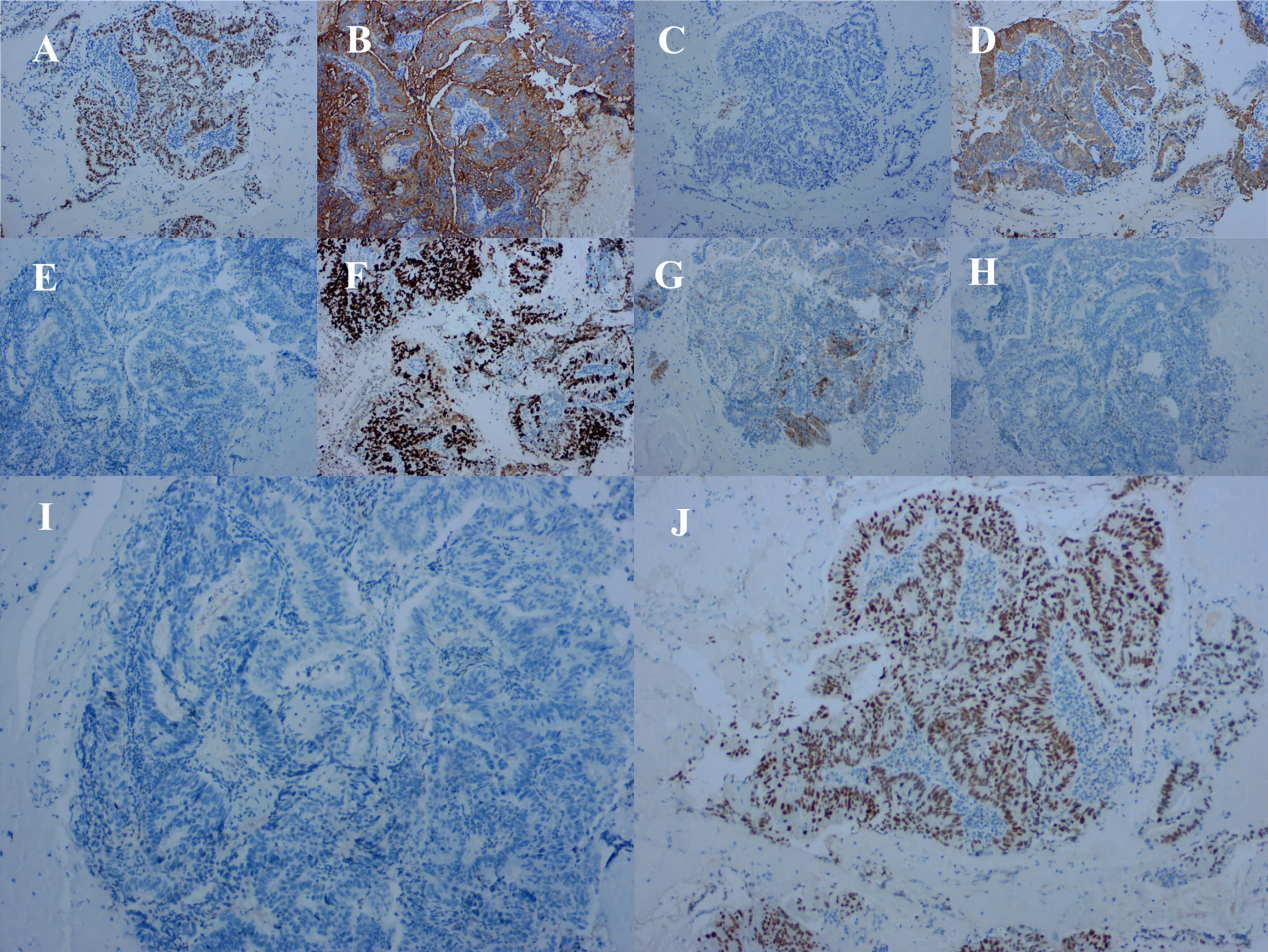
Immunohistochemical staining of tumor cells for **(A)** caudal-related homeobox 2 (positive), **(B)** carcinoembryonic antigen (positive), **(C)** cytokeratin (CK) 7 (negative), **(D)** CK20 (positive). **(E)** estrogen receptor (negative), **(F)** Ki-67 (approximately 70% positive), **(G)** P16 (partially positive), **(H)** P53 proteins (negative), **(I)** progesterone receptor (negative), and **(J)** special AT-rich sequence-binding protein 2 (positive).

Because the primary origin of the tumor was unclear and the vaginal segment of the cervix was narrow, hysteroscopic exploration was initially performed during which the operator required several attempts to penetrate the cervical opening and required the use of ultrasonography to explore the uterine cavity. Endometrial thickening was observed in the cervical opening, and the cervical canal itself was thickened and fragile. Postoperative pathology of cervical and endometrial tissue revealed highly differentiated adenocarcinoma consistent with an intestinal epithelial origin (**Figure 3**). Immunohistochemistry revealed positive staining for carcinoembryonic antigen, Ki-67 (approximately 70%), cytokeratin (CK) 20, caudal-related homeobox 2, and special AT-rich sequence-binding protein 2; partially positive staining for p16 protein; and negative staining for estrogen receptor, progesterone receptor, p53 proteins, and CK7 (**Figure 4**). Additionally, the tissues were positive for the mismatch repair proteins MLH1, PMS2, MSH2, and MSH6.

Evidence from the pathological examination indicated that the tumor in the cervix and endometrium originated from the intestine. Given the patient’s history of sigmoid colpoplasty 18 years prior, the primary lesion was presumed to have originated in the sigmoid colon-derived neovagina. Considering the patient’s current pelvic anatomical disorder, severe tissue adhesions, and the early stage of the localized tumor, we planned to administer neoadjuvant treatment to reduce the tumor stage followed by combined gynecological/colorectal surgery to remove the cancerous tissue. However, the patient refused surgery and was treated instead with four cycles of oxaliplatin plus capecitabine (the CapeOX chemotherapy regimen). Three months after chemotherapy, the patient was followed up once, and enhanced pelvic magnetic resonance imaging indicated that the cervical mass was slightly larger than before, and the posterior irregular mass was smaller than before. We decided that the patient was not suitable for continuing chemotherapy and decided to undergo surgery, but the patient still refused surgery.

## Discussion

Primary vaginal dilation is successful for more than 90–96% of patients (2), whereas colpoplasty is appropriate for patients who have failed dilation or who have voluntarily opted for surgery. The most common colpoplasty methods include autologous grafts, such as partial-thickness or full-thickness skin grafts; peritoneal, buccal mucosa, or small bowel grafts; allogeneic human amniotic membrane insertion; or synthetic materials. In our hospital, sigmoid colpoplasty is the most common procedure because of its high success rate and the similarity to the normal vagina with respect to shape and function. Complications associated with this procedure include dyspareunia, stenosis of the vaginal opening (3), intestinal obstruction, anastomotic fistula, neovaginal prolapse, colitis, hereditary polyposis, and adenocarcinoma (4). Intestinal neovaginal carcinoma is a rare complication; we retrieved relevant 13 case reports from the literature. All cases were adenocarcinoma (**Table 1**). In contrast, squamous cell carcinomas are more prevalent in patients who undergo skin graft colpoplasty. As such, the type of cancer appears to be dependent on the origin of the tissue used for neovaginal replacement; our case report focuses on the former.

**Table 1:** Previous reports of malignant lesions in patients who underwent sigmoid colpoplasty.

Author/Year	Primary diagnosis	Vaginoplasty tissue	Age at vaginoplasty/Cancer discovery	Symptoms	Physical examination	Medical history	Dignosis	Treatment
Ritchie 1929 (5)	Pelvic chronic abscess/MRKH	Ileum	13/26	Abdominal pain and vaginal bleeding	Vaginal mass	Unknown	AC	Radiation
Lavand 1938 (6)	MRKH	Ileum	18/33	Vaginal bleeding, fistula	Vaginal nodule	Unknown	AC	Radiation
Andryjowicz et al., 1985 (7)	Vaginal carcinoma	Cecum	39/42	Inspection findings	Irregularity of neovagina mucosa	Irradiation	AC	Operation
Auber et al., 1989 (8)	MRKH	Ileum	20/59	Vaginal bleeding	Vaginal nodule	Unknown	AC	Operation and radiation
Borruto et al., 1990 (9)	No information	Ileum	21/60	Vaginal bleeding	Vaginal mass	Unknown	AC	Radiation
Ursic-Vrscaj et al.1994 (10)	Cervical cancer	Sigmoid	36/58	Vaginal bleeding	No information	Unknown	AC	Operation
Hiroi et al.2001 (11)	MRKH	Sigmoid	23/53	Vaginal bleeding	Vaginal mass	Unknown	AC	Operation and radiation
Schouten et al.2005 (12)	Botryoid sarcoma	Sigmoid	28/45	Abdominal pain and vaginal bleeding	Vaginal mass	Radiation therapy/remnant sigmoid adenocarcinoma	AC	Operation and chemotherapy
Lambert et al.2013 (13)	Cloaca	Sigmoid	7/30	Vaginal bleeding and urinary retention	Urethral mass and vaginal polyps	Unknown	AC	Chemotherapy(Refused surgery)
Kita et al.2015 (14)	MRKH	Sigmoid	27/67	Vaginal bleeding	Vaginal mass	Unknown	AC	Operation and adjuvant therapies
Bogliolo et al., 2015 (15)	Partial vaginal agenesis with recto-vaginal fistula	Rectosigmoid	13/61	Vaginal bleeding	Vaginal neoplasms	(Age 30)Pelvic abscess underwent operation	AC	Operation and radiation
Yamada et al., 2018 (16)	MRKH	Sigmoid	23/76	Vaginal bleeding	Residual vaginal and sigmoid anastomotic lesions	(Age 45)Uterine myoma underwent hysterectomy	AC	Operation
Christophe et al.2021 (17)	MRKH	Sigmoid	27/67	Vaginal bleeding	Budding part in the neovaginal lumen	(Age 48) Breast cancer	AC	Operation and chemotherapy

MRKH, Mayer-Rokitansky-Küster-Hauser Syndrome; AC, adenocarcinoma.

The etiology of neovaginal adenocarcinoma is unknown and is likely associated with inflammation, human papillomavirus (HPV) infection, the tissue microenvironment, and lifestyle factors. Sterpetti et al. (18) found that age, as well as the clinical and histological characteristics, of patients with neovaginal cancer is similar to that of patients diagnosed with sporadic colorectal cancer in the general population; moreover, the prevalence rates of both are similar. Bacterial overgrowth, infection, and inflammation may be risk factors leading to chronic or acute inflammation and damage to the colonic mucosa, which can eventually lead to carcinoma. Alaniz et al. (19) also suggested that neovaginal inflammatory bowel disease is a risk factor for precancerous and malignant lesions in the neovagina. Two patients listed in **Table 1** (15, 17) were found to have evidence of current or previous active inflammation. Similarly, our patient had a history of pelvic inflammatory disease, and evidence of intestinal inflammation was found during her gastroenteroscopy.

Diversion colitis is a nonspecific inflammatory process that occurs in segments of the colorectum that are diverted from the fecal stream. This diversion results in a deficiency of short-chain fatty acids (from the contents of the colon), which are essential nutrients for the colonic epithelium. Short-chain fatty acids play an important regulatory role in cell proliferation and differentiation, and their deficiency may alter the cellular environment. In a study of vaginal endoscopic findings in 34 patients with sigmoid neovaginas, 31 of them (91%) showed evidence of inflammation on colposcopy, although the majority of those with inflammatory changes were asymptomatic (20). Although it is possible that diversion colitis may promote the development of adenocarcinoma, there remains a lack of endoscopic and histopathological data to support this.

Increasing evidence also suggests that HPV infection plays a potential role in the pathogenesis of colorectal cancer (21); however, the etiology of this disease is multifactorial and HPV may play a role in only certain subtypes of it. Recent meta-analyses (22, 23) showed a significantly increased correlation between colorectal cancer and the presence of HPV infection. Rall et al. (24) found that the rate and type of vaginal HPV infection in patients with MRKH syndrome who underwent colpoplasty were comparable to those in the general population, which indicates that the surgically produced vaginal epithelium was normal in terms of HPV susceptibility.

The sigmoid neovagina may experience minor trauma from repeated friction during dilation and sexual intercourse, which may lead to persistent granulation tissue, chronic infection, and inflammation; this also increases the risk of malignant lesions. Yamada et al. (16) found that carcinomas of sigmoid neovaginas tend to occur near the site of anastomosis. Other than the neovagina, secondary malignant tumors of the ileal neobladder also occur at the ileoureteral anastomosis (25), suggesting that anastomosis between the bowel and other organs may increase the risk of cancer. Chemical irritation from semen and urine may contribute to malignancy. Coincidentally, our patient developed the carcinoma at the exact site of the anastomosis of the sigmoid colon and cervix, which supports the notion that the risk of lesions at the site of anastomosis is higher than that at other locations; this should be investigated further.

**Table 1** summarizes 13 patients with intestinal neovaginal malignancies, all of which were adenocarcinomas. These patients were diagnosed 3–53 years after surgery, with a mean time to presentation of 29 years and a minimum time of 3 years after surgery. The majority of patients (92%) presented with symptoms of vaginal bleeding, and examination revealed a mass at the neovagina (70%); a biopsy confirmed the diagnosis of neovaginal cancer. Our patient also complained of vaginal bleeding with lower abdominal pain; however, owing to the narrow vaginal segment of the cervix, no vaginal mass was found on examination. The diagnosis of adenocarcinoma was confirmed only after a hysteroscopic biopsy, which was found to be of intestinal origin and invaded the cervix and endometrium.

Colposcopy and biopsy are the principal tools for confirming a diagnosis of neovaginal cancer, and modern imaging techniques, including transvaginal ultrasonography, magnetic resonance imaging, computed tomography, and positron emission tomography/computed tomography are often used to assess the extent of tumors and to develop surgical plans. After biopsy or surgery, immunohistochemistry is essential for confirming the diagnosis; investigating the presence of estrogen receptor, progesterone receptor, CK7, CK20, and caudal-related homeobox 2 can be used to differentiate colorectal cancer from endometrioid adenocarcinoma.

There is no consensus on the standard of care for neovaginal cancer, and previously published reports have described the use of highly individualized treatments. Three patients were treated with radiotherapy, one refused surgery and opted for chemotherapy, and the remaining nine opted for surgery. The follow-up periods of all the patients were relatively short (between 6 months and 2 years), and only the patient reported by Ritchie et al. was known to have died 6 months after radiotherapy. As such, surgery is clearly a feasible option, although additional radiotherapy or chemotherapy may be required afterward depending on the extent of tumor invasion. Adhesions are an important factor to consider when planning surgery, and it is essential for the gynecologist perform the surgery together with a gastroenterologist or surgeon. In our patient, we discovered strong adhesions between the neovagina and rectum and bladder, which led us to judge that resection of the neovagina alone would be difficult. We planned to perform adjuvant chemotherapy followed by total pelvic dissection to achieve complete resection with microscopically negative incision margins (R0); however, because the patient refused surgery, we only performed four cycles of neoadjuvant chemotherapy. Three months after chemotherapy, the patient was followed up once, and enhanced pelvic magnetic resonance imaging indicated that the cervical mass was slightly larger than before, and the posterior irregular mass was smaller than before. We decided that the patient was not suitable for continuing chemotherapy and decided to undergo surgery, but the patient still refused surgery.

In conclusion, patients with a history of colpoplasty should undergo long-term follow-up post-reconstruction. The most common first symptom of neovaginal cancer is discharge or bleeding; such symptoms (whether recent or long past) warrant attention and thorough pelvic examination (26). Colposcopy and biopsy are essential for evaluating the lesion and determining the cause of bleeding. Immunohistochemistry can reveal the nature of the primary lesion, and imaging techniques can assess the extent of tumor spread. Surgery is the treatment of choice and can be combined with radiotherapy or chemotherapy. Further studies on the mechanism of neovaginal carcinogenesis will provide guidance on long-term management and improve the quality of life of patients after colpoplasty, and provide more choices for patients.

## Author contributions

XFS, managed and tracked patient outcomes, collected relevant clinical data, wrote, and submitted manuscripts. XXM, summarized important clinical data and revised the manuscript. BXL, made all the diagrams in the article and revised the manuscript. YW, a biopsy was performed that helped confirm the final diagnosis. All authors contributed to this article and approved the submitted version.

## Conflict of interest

The authors declare that the research was conducted in the absence of any commercial or financial relationships that could be construed as a potential conflict of interest.

## Publisher’s note

All claims expressed in this article are solely those of the authors and do not necessarily represent those of their affiliated organizations, or those of the publisher, the editors and the reviewers. Any product that may be evaluated in this article, or claim that may be made by its manufacturer, is not guaranteed or endorsed by the publisher.
